# *MUTYH* and *KLF6* gene expression fluctuations in tumor tissue and tumor margins tissues of colorectal cancer

**DOI:** 10.1186/s43046-022-00158-9

**Published:** 2022-12-05

**Authors:** Hoora Naebi, Ahmadreza Bandegi, Fereshteh Talebinasab, Pirouz Samidoust, Seyedeh Elham Norollahi, Sogand Vahidi, Ali Akbar Samadani

**Affiliations:** 1 Department of Biochemistry, Faculty of Medicine, Semnan University of Medical Sciences, Semnan, Iran; 2 Student Research Committee, Semnan University of Medical Sciences, Semnan, Iran; 3 Cancer Research Center and Department of Immunology, Semnan University of Medical Sciences, Semnan, Iran; 4 Razi Clinical Research Development Unit, Guilan university of medical Sciences, Rasht, Iran; 5 Medical Biology Research Center, Kermanshah University of Medical Sciences, Kermanshah, Iran; 6 Guilan Road Trauma Research Center, Guilan University of Medical Sciences, Rasht, Iran

**Keywords:** Colorectal cancer, *KLF6*, *MUTYH*, Gene expression

## Abstract

**Background:**

Colorectal cancer (CRC) is one of the most important cancers in the world, and its prevalence varies depending on the geographical area. Genetically, tumor regeneration in CRC as a multi-step process involves activating mutations in protocogenes and losing the function of tumor suppressor genes as well as DNA repair and recovery genes. Occur in this way, our goal was to investigate the expression of *KLF6* genes as a tumor suppressor and *MUTYH* involved in the DNA repair process in colorectal cancer.

**Methods:**

This research was conducted during the years 2019–2018 in Razi Hospital, Rasht. The subjects included 30 tumoral and 30 non-tumoral tissues of colorectal cancer and 20 healthy controls. The real-time PCR method was used to investigate the gene expression. For data analysis by SPSS, parametric statistical tests ANOVA and *T* test and regression analysis were used and *p* value values less than 0.05 were considered significant.

**Results:**

The expression of *KLF6* gene in tumoral tissues showed a significant decrease compared to non-tumoral tissues (*P* = 0.04). Also, the expression of *MUTYH* gene in tumor tissue showed a significant decrease compared to non-tumoral (*P* = 0.02) and this decrease in *MUTYH* gene expression had a significant relationship with increasing tumor stage (*P* = 0.01).

**Conclusion:**

These findings suggest that decreased expression of *KLF6* and *MUTYH* genes in the study population has a significant relationship with colorectal cancer and can be considered as tumor marker in diagnostic purpose.

## Background

Colorectal cancer (CRC) is one of the leading causes of death worldwide and is one of the most common malignancies of the gastrointestinal tract, with high mortality rate deaths annually [1]. In general, the main cause of colorectal cancer is genetic (genetic mutation) and epigenetic changes [2]. Most of these changes occur on the genes of oncogen, tumor suppressor, and DNA repair gene. Therefore, the suppression of tumor suppressor genes and the activation of oncogenic genes play a key role in carcinogenesis and tumorigenesis of CRC [3].

The *MUTYH* gene is located on the short arm of chromosome 1 with about 11.2 kg and has 16 exons. This gene encodes a protein with 535 amino acids, a type of glycosylase, and is involved in DNA repair [4]. In the body, the active species of oxygen and nitrogen form 8-oxoG in the genome. *MUTYH* reduces the conversion of G: C to A: T by removing adenine from the open 8-oxoG [5]. Mutations in the *MUTYH* gene increase the open-to-oxoG gene, which is abundant in the APC, KRAS, PIK3CA, FAT4, Tp53, FAT1, AMER1, KDM6A, SMAD4, and SMAD2 genes associated with CRC [6].

In this account, another important genes in our project was *KLF6* (Krüppel-like factor 6). Changes in the expression of the *KLF6* gene also play an important role in the development of cancer. Consequently, the function of this gene is suppressing the tumor and is located on the 10p15 chromosome. The occurrence of mutations in this gene is involved in the development of some types of tumors, so that the association of mutations in this gene with cancers such as colorectal [7], malignant glioma [8], nasopharyngeal carcinoma [9], breast cancer [10], gastric cancer [11], and prostate cancer [12] have been reported.

Importantly, *KLF6* is a fingerprint transcription factor that is expressed in all cells and is part of the growing KLF family. The KLF family is widely involved in the transmission of signals related to growth, cell proliferation, development, apoptosis, as well as angiogenesis. The performance of the natural *KLF6* species increases the P21 inhibitor in a type independent of TP53 and inhibits growth. In fact, this protein interrupts the function of cyclin-dependent kinase D1 and stops the cell cycle in the G1 phase. *KLF6* protein also acts as a cell proliferative inhibitor via pro-ketoprotein [13, 14].

Considerably, the importance of this cancer from a molecular point of view is necessary for the diagnosis, and follow up in treatment and prevention. In this study, the expression of *KLF6* and *MUTYH* genes in patients with CRC was studied for the first time in an Iranian population in this area.

## Methods

### Sampling

This research was conducted during 2019 in Razi Hospital in Rasht, Iran. A total of 50 people (30 people with colon cancer and 20 healthy people as a control sample) were screened. After obtaining written consent from all the patients, specimens were prepared directly in the surgery room and we did not use biopsies of colonoscopy. It means we just used surgical tissues (tumoral and non-tumoral tissues). The diagnosis of colorectal cancer was made by a pathologist based on clinical and histopathological findings.

In the surgery rooms, the tissues were put in the liquid nitrogen tank and some of the were pun in the RNA latter solution. The samples were transferred to the laboratory and stored at – 70 °C for more investigation.

### RNA extraction

The samples were first grinded by liquid nitrogen and RNA was extracted according to the Kit protocol (BIONEER, serial number K-3090). To evaluate the quality of extracted RNA, and all the RNA were electrophoresed on, 1.5% agarose gel and spectrophotometer nanodrap were used in order to control the quality of the extracted RNA.

### cDNA synthesis and real-time PCR

Then, cDNA was created according to Kate’s agenda (Revert AID First Strand cDNA synthesis Thermo scientific with serial number K1621). Initially, 5 μg of sample RNA and 1 μl of Random hexamer primer were delivered to 12 μl using nuclease-free water. It was then placed at 95 °C for 5 min to eliminate secondary RNA structures.

In the next step, the second mix included 4 μl Reaction buffer 5X, 1 μl Ribolock RNase Inhibitor, 2 μl dNTP mix 10 mM and 1 μl Revert Aid M-mulV RT were combined. Subsequently, 8 μl of this material were added to the contents of the first stage and the final volume was increased to 20 μl.

The cDNA compounds were then placed at 42 °C for 60 min and then at 25 °C for 5 min for randomized rheumatic primer activity. Finally, in order to inactivate the RT enzyme, microtubules containing the sample were placed at 70 °C for 5 min. Primers were designed using NCBI software (Table 1).

**Table 1:** Forward and reverse sequences of *KLF6* and MUYTH genes

Gene	Forward	Reverse	bp
**KLF6**	CGCTGCCGTCTCTGGAGGAGT	CAGGGCTCGCTCTGGAGGTAAC	73
**MUYTH**	CTTCCGAGGGAGCCTGCTAAG	CATCTCATCTTCTGCCCGTCTTC	79

The resulting of cDNA was used as a model for qRT-PCR using cybergreen (Thermo scientific maxima SYBR Green/Rox qPCR Master Mix with serial number K0221). Amplification was performed using a Bio-Rad Real-time PCR set.

### PCR cycle program

The reaction of the target samples took place in 41 cycles and each cycle took place in two stages. The first step was to separate the two strands (Denaturation) for 15 seconds at 95 C, and the second step was to connect the primers (Annealing) and extend (Extension) at 60 C for 60 seconds. In this reaction, GAPDH was used as internal control and healthy samples were used as calibration to reduce the incidence of error in this study.

### Statistical analysis

The data were analyzed using the SPSS software version 22. *T* test, ANOVA, and regression analysis were used to analyze the expression of these genes. The fold change method was used to compare the expression of the studied genes with the healthy samples. The rate of change in the expression of these genes was normalized with the internal control gene and then the rate of change in the expression of the genes in the control or healthy sample was investigated. Significant values of *P* < 0.05 were considered.

## Results

The characteristics of patients and their clinical and pathological characteristics are shown in Table 2.

**Table 2:** Patient profile

Characteristics	Total (*N* = 30) Patients (%)
Gender	
Female	12 (40)
Male	18 (60)
Age	
< 60 years	21 (70)
≥ 60 years	9 (30)
Stage	
I	1 (3.3)
II	16 (53.3)
III	11 (36.7)
IV	2 (6.7)
Grade	
Well differentiated	8 (26.7)
Moderate differentiate	10 (33.3)
Poorly differentiate	11 (36.7)
Undifferentiated	1 (3.3)
LM	
Yes	22 (73.3)
No	8 (26.7)
DM	
Yes	21 (70)
No	9 (30)

**Fig. 1:**
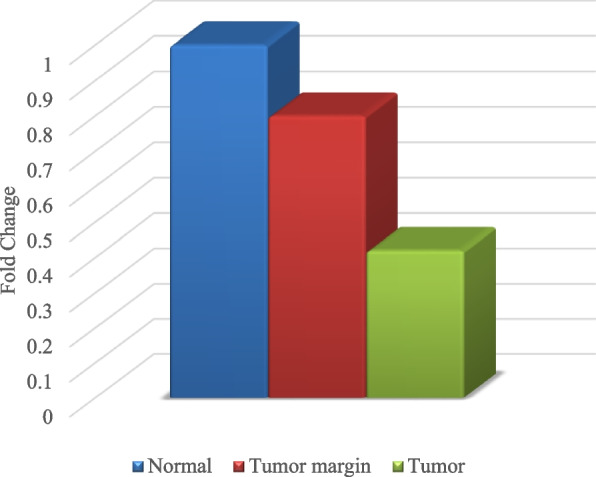
Comparison of fold change in KLF6 gene between tumor tissue and tumor margin and normal tissue

In this study, fold change method was used to investigate the changes in the expression of *MUTYH* and *KLF6* genes in polyps of different intestinal regions. Our results illustrated that the expression level of *KLF6* decreased in more than 73% of tumoral specimens compared to non-tumoral specimens (*P* < 0.05) (Fig. 1).

**Fig. 2:**
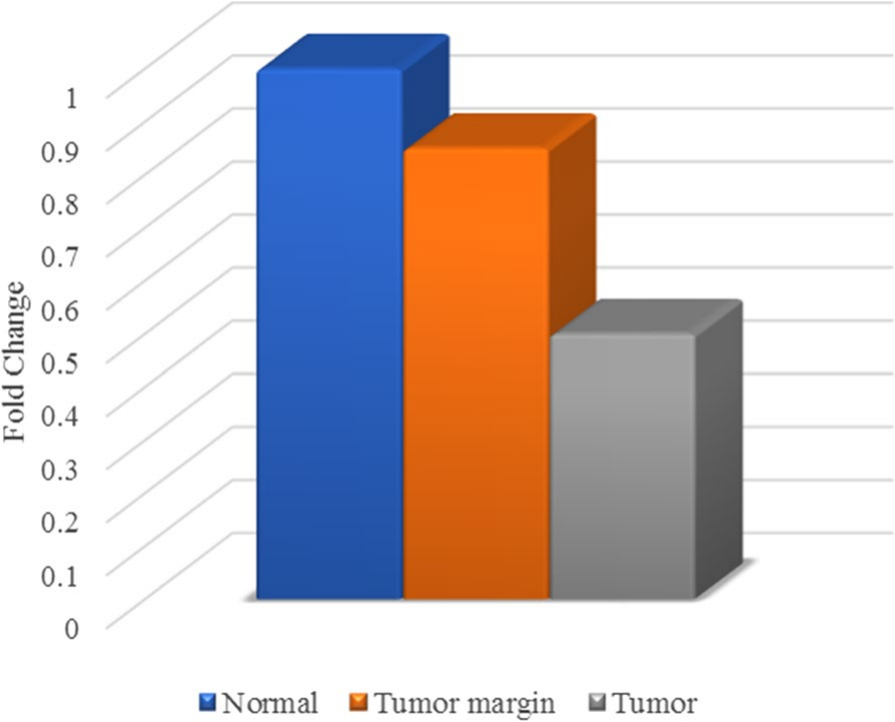
Comparison of fold change in the MUTYH gene between tumor tissue and tumor margin and normal tissue

**Fig. 3:**
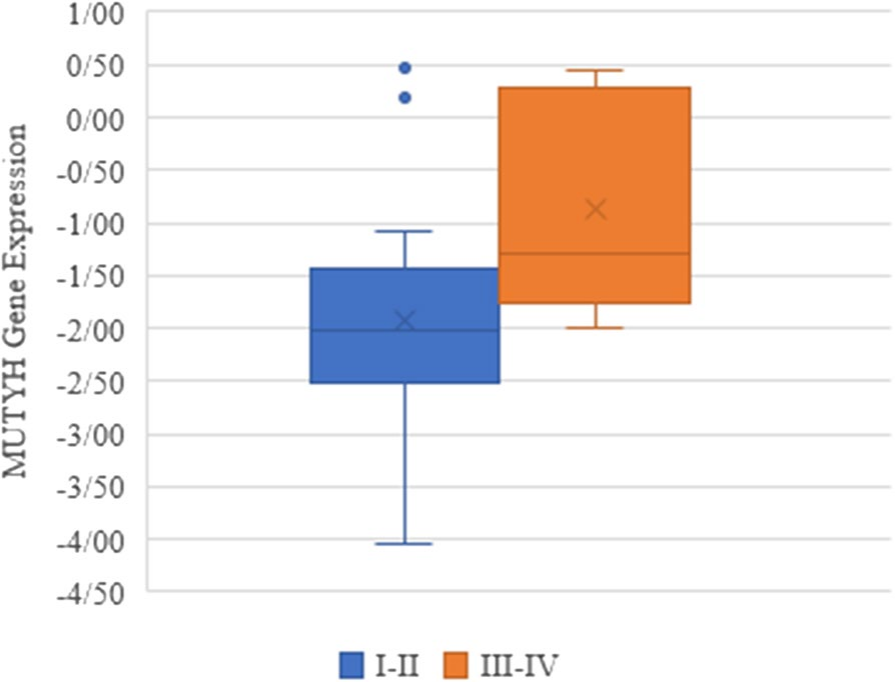
The relationship between MUTYH gene expression and tumor stage

**Fig. 4:**
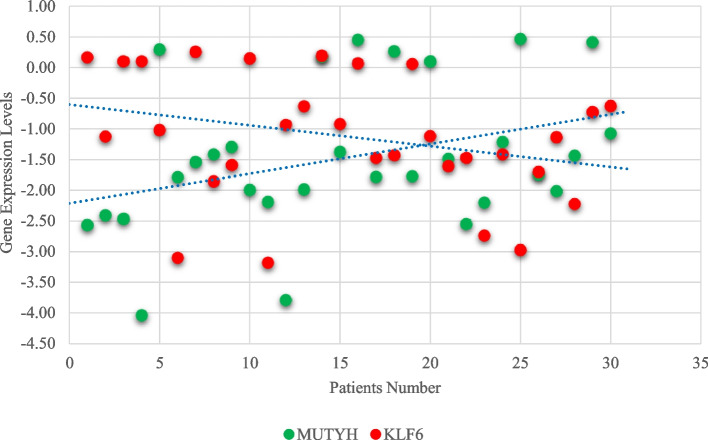
Distribution of relative expression of KLF6 and MUTYH genes in patients with colorectal cancer

Considerably, *MUTYH* expression levels were significantly reduced in more than 76% of tumor specimens compared with non-tumor specimens (*P* = 0.02) (Fig. 2). Our results also indicated that a decrease in *MUTYH* expression had a significant relationship with tumor stage (*P* = 0.01). Remarkably, with increasing the tumor stage, its expression decreased further (Fig. 3). The expression of *KLF6* (*P* = 0.04) and *MUTYH* (*P* = 0.02) genes simultaneously decreased significantly in colorectal cancer samples compared to non-tumor samples (Fig. 4) (Table 3).

**Table 3:** Clinicopathological features of two genes, KLF6 and MUYTH, in patients with CRC

	*KLF6*		*P* value	*MUTYH*		*P* value
Tumor stage	↓/−	↑	0.9	↓/−	↑	0.01
III	11	6		15	2	
III–IV	11	2		8	5	
Tumor grade			0.4			0.2
I–II	12	6		13	5	
III–IV	10	2		10	2	
LM			0.06			0.8
Yes	15	7		17	5	
No	7	1		6	2	
DM			0.9			0.1
Yes	17	4		16	5	
No	4	5		7	2	

*LM* lymph node metastasis, *DM* distance metastasis

## Discussion

Colorectal cancer is considered to be one of the most important cancers in the world, and its prevalence varies depending on the geographical area. Genetically, tumor regeneration in CRC is a multi-step process that involves activating mutations in protocogens and losing the function of tumor suppressor genes. In addition, DNA repair and recovery genes play an important role in tumor conservation by maintaining the integrity of the genome [3]. In this way, the results of our project were so changeable and the expression of the *KLF6* gene, as a tumor suppressor, and the *MUTYH* gene, which was involved in the DNA repair process; indicated a positive relationships in the carcinogenesis of CRC [6, 7].

Meaningly, the present study hypothesized that changes in the expression of *KLF6* and *MUTYH* genes at the RNA level on colon polyps could be a good factor in assessing the malignancy of polyps and ultimately early detection of colorectal cancer.

In a study by REEVES et al., a decrease in *KLF6* expression may have contributed to the development of colorectal cancer and interestingly, it was the similar results, like in our project [7]. Cho et al. measured the *KLF6* gene expression pattern in 123 samples of colorectal cancer tissue by immunohistochemistry and stated that a decrease or absence of *KLF6* expression may be a primary or common occurrence in colorectal cancer [13].

In another study, a small role for mutations in the *KLF6* gene in CRC was reported in French patients [15]. Zhang et al. identified the role of SV2, one of the *KLF6* isoforms, in CRC, and stated that it plays the role of tumor suppressor by preventing cell proliferation, stopping the cell cycle, and inducing apoptosis in CRC, which may increase. It was also observed that the expression level of mRNA (*KLF6-sv2*) in CRC decreased compared to normal tissue [16]. However, in the present study, it was found that reducing the expression of *KLF6* gene in tumor tissue was significant compared to non-tumor tissue.

As mentioned about the function of the *KLF6* gene, the natural species *KLF6* increases P21 inhibitor in an independent type of TP53 and inhibits growth. In fact, it inhibits the function of cyclin-dependent kinase D1 and stops the cell cycle in stage G1. Conspicuously, *KLF6* also acts as a inhibitor of cell proliferation by acting against pro -coprotein C-Jun [13, 14]; therefore, it can act as a tumor suppressor protein, resulting in reduced expression of cancer.

KUNO et al. reported that somatic changes in the *MUTYH* gene, like other DNA repair genes, are rare in colorectal cancer, and that unknown mutations and epigenetic changes in the prometer area of the *MUTYH* gene may contribute to the disease [17].

In a study by Takao et al., some patients suspected of having inherited colorectal polyposis in Japan had two-allele or single-allele *MUTYH* variants, and the common Caucasian *MUTYH* variants were not sensitive to Japanese patients [18].

A study by Nascimento et al. showed that tissue expression of the *MUTYH* gene in Brazilian patients decreased with the progression of colorectal cancer [19]. Dumanski et al. also found that a mutation in p. (Gly369Asp) in *MUTYH* resulted in familial or mononuclear small bowel neuroendocrine tumors [20].

In the present study, a decrease in the expression of *MUTYH* gene in tumoral tissue compared to non-tumor tissue showed significant changes, which can confirm the performance of *MUTYH* gene protein. Because, as mentioned, the protein made by this gene can act as a base excision repair (BER). And if the gene’s expression is reduced, its function in the cell, and more generally in the tissue, is reduced, and to a lesser extent it can remove the error, which results in the malfunction of the tumor.

Our findings also suggest that a reduction in the expression of this gene may be significantly related to the stage of tumoral tissue.

## Conclusion

In summary, our study found that a decrease in the expression of the *MUTYH* and *KLF6* genes was significantly associated with colorectal cancer and could be considered as a tumor marker tumor for colorectal cancer. They may also be used for gene therapy in the future.

## Data Availability

The datasets used and/or analyzed during the current study are available from the corresponding author on reasonable request.

## Abbreviations

CRC: Colorectal cancer
*KLF6*: Krüppel-like factor 6
BER: Base excision repair
